# Apparent Diffusion Coefficient Values and Dynamic Contrast-Enhanced Magnetic Resonance Perfusion are Potential Predictors for Grading Meningiomas

**DOI:** 10.7150/ijms.75092

**Published:** 2022-07-18

**Authors:** Sri Andreani Utomo, Abdul Hafid Bajamal, Yuyun Yueniwati, Irfan Deny Sanjaya, Dyah Fauziah

**Affiliations:** 1 Doctoral Program of Medical Science, Faculty of Medicine, Universitas Airlangga, Indonesia; 2 Department of Radiology, Faculty of Medicine, Universitas Airlangga, Indonesia; 3 Department of Neurosurgery, Faculty of Medicine, Universitas Airlangga, Indonesia; 4 Department of Radiology, Faculty of Medicine, Brawijaya University, Malang, Indonesia; 5 Department of Anatomical Pathology, Faculty of Medicine, Universitas Airlangga, Indonesia

**Keywords:** Grading meningioma, ADC value, DCE-MRI

## Abstract

**Purpose**: Distinguishing between high-grade and low-grade meningiomas might be difficult but has high clinical value in deciding precise treatment and prognostic factors. Magnetic resonance imaging (MRI) using apparent diffusion coefficient (ADC) values and dynamic contrast enhancement (DCE) may have a significant role in capturing such complexities.

**Methods**: Data from our hospital database on meningioma patients from January 2020 to December 2021 were obtained. The MRI results of all patients were evaluated for mean ADC value and DCE parameters, including time-signal intensity curves (TIC), maximum signal intensity (SImax), time to maximum signal intensity (Tmax), maximum contrast enhancement ratio (MCER), and slope.

**Results**: In this retrospective analysis, 33 individuals were included. Twenty-eight (84.8%) patients were pathologically diagnosed with low-grade meningioma and five (15.2%) patients with high-grade meningioma. There is a crossover between high- and low-grade meningiomas in conventional MRI. Tumor size, location, shape, necrotic/cystic changes, peritumoral edema, and enhancement patterns did not differ substantially between groups (*p* = 0.39, 0.23, 0.28, 0.57, 0.56, and 0.33, respectively). The mean ADC and Tmax values of high-grade meningiomas were substantially lower than those of low-grade meningiomas (*p* = 0.002 and 0.02, respectively). An optimal cut-off of 0.87 × 10^-3^ mm^2^s^-1^ for the mean ADC value (area under the curve [AUC] = 0.94, sensitivity = 80%, specificity = 92.8%) and 42 s for Tmax (AUC = 0.84, sensitivity = 80%, specificity = 89.3%) was suggested. High-grade meningiomas had significantly higher TIC, SImax, MCER, and slope than low-grade meningiomas (*p* = 0.004, < 0.001, 0.01, and 0.001, respectively). Type IV TIC had a sensitivity of 80% and specificity of 89.3% in distinguishing high-grade meningiomas from low-grade meningiomas. Optimal cut-offs of 940.2 for SImax (AUC = 0.98, sensitivity = 80%, specificity = 96.4%), 245% for MCER (AUC = 0.94, sensitivity = 80%, specificity = 85.7%), and 5% per second for slope (AUC = 0.97, sensitivity = 80%, specificity = 96.4%) were estimated.

**Conclusion**: The ADC value and DCE-MRI parameters (TIC, SImax, Tmax, MCER, and slope) are potential predictors for separating high-grade from low-grade meningiomas.

## Introduction

Meningiomas are the most frequent primary intracranial tumors and originate from arachnoid capcells, contributing to over 30% of all intracranial neoplasms in adults. Meningiomas are classified by the World Health Organization (WHO) into three grades and subdivided into 15 histological subtypes. WHO grade I meningiomas are also referred to as low-grade meningiomas (LGMs), while WHO grade II/II meningiomas are high-grade meningiomas (HGMs). Although most meningiomas are LGMs, representing approximately 80-90% of all meningiomas, HGMs are more aggressive, with a higher risk of recurrence 1. The clinical outcomes and survival rates of HGMs are worse than those of LGMs. The grading of meningiomas has a high clinical impact on deciding the precise treatment plan and improving prognosis; therefore, a diagnostic tool that can distinguish between the two groups of meningiomas is desirable.

Currently, the presurgical diagnosis of meningiomas mostly depends on magnetic resonance imaging (MRI). Distinguishing HGMs from LGMSs may be difficult using conventional MRI as no specific feature of conventional MRI can dependably predict meningioma grades; however, advanced MRI may be helpful 2. Diffusion-weighted imaging (DWI) and apparent diffusion coefficient (ADC) values are generally used for brain tumors. ADC values are often useful for tumor grading as the velocity of diffusion of water molecules is inversely related to the degree of tumor cell density and membrane integrity 3. Previous studies have shown that the ADC value can be used to differentiate between LGMs and HGMs 3-7.

Perfusion MRI has been widely used to evaluate neuro-oncology cases because of the strong correlation between tumor neoangiogenesis and grading. Currently, the most widely utilized perfusion MRI protocol for brain tumors is dynamic susceptibility contrast (DSC), which allows for the calculation of the relative cerebral blood volume. However, in DSC-MRI, the location of regions of interest (ROIs) can be impeded by susceptibility artifacts at tissue interfaces or in the skull base region, which is a common location for meningiomas 8. Dynamic contrast enhancement (DCE) is another perfusion MRI protocol that allows for the quantitative measurement of absolute tissue perfusion without being affected by the magnetic field 9. Previous studies have suggested DCE-MRI as a tool to differentiate between LGMs and HGMs.

The quantitative parameters usually obtained in DCE-MRI are the rate constant (*K*ep), volume transfer constant (*K*trans), mean plasma volume (*V*p), and extracellular volume (*V*e) 9-11. The gadolinium contrast agent passes through the microvasculature and leaks from the intravascular compartment to the extracellular extravascular space (EES) via passive diffusion, modifying tissue signal intensity. *K*trans refers to the volume transfer constant of gadolinium from blood plasma to the EES. *K*ep refers to the time constant of gadolinium transfer from the EES back into the intravascular space. *V*p is plasma volume per unit tissue volume, while *V*e is EES volume per unit tissue volume 12-14.

However, to our knowledge, the evaluation of semiquantitative DCE-MRI parameters, such as time-signal intensity curve (TIC), maximum signal intensity (SImax), time to maximum signal intensity (Tmax), maximum contrast enhancement ratio (MCER), and slope, is still lacking. Semiquantitative DCE-MRI parameters also depict hyperdynamic patterns that can be utilized to differentiate and grade tumors.

Therefore, the purpose of this research was to investigate the ability of conventional, DWI, and DCE-MRI to distinguish HGMs from LGMS based on the mean ADC value, TIC, SImax, Tmax, MCER, and slope. The findings of this research may be useful in improving the diagnosis and treatment of meningiomas.

## Methods

### Patient Characteristics

This study was revised and certified by the ethics committee of our hospital (letter of exemption Ref. No.: 0526/ LOE/ 301.4.2/VII/ 2021). Patients with meningioma who underwent MRI examination at the hospital's Department of Radiology between January 2020 and December 2021 were consecutively enrolled in this retrospective study. The inclusion criteria were as follows: 1) patients who underwent preoperative MRI examination, including DWI and DCE-MRI; 2) patients who underwent surgical resection at the Department of Neurosurgery; and 3) patients with histopathologically confirmed meningiomas and WHO grading. Patients with substantial motion artifacts and poor image quality were excluded. Finally, 33 patients were enrolled in this research.

### Image Acquisition

Head MRI examinations were performed using a 3.0T scanner (MAGNETOM Skyra, Siemens, Erlangen, Germany) and head coil according to the standard operating procedure at our hospital. MRI examinations included T1- and T2-weighted imaging, T2 fluid-attenuated inversion recovery, contrast-enhanced T1-weighted imaging, DWI, and DCE-MRI. DWI was performed before contrast agent administration using TR/TE 4430-6640/55-76 ms, a field of view of 250 mm, b-values of 50 s/mm^2^ and 800 s/mm^2^, a slice thickness of 3.5 mm, a slice gap of 1 mm, and an acquisition matrix (voxel) of 115 × 128. ADC maps were automatically produced using a default software package. The contrast agents were administered intravenously with gadoteric acid (0.1 mmol/kg) and injected at 2 mL/s, followed by 20 mL of normal saline at a similar rate. DCE was then achieved using 3D T1, TR/TE 5.48/1.97 ms, 30-40 slices/slab, a flip angle of 15°, a slice thickness of 3.5 mm, a slice gap of 1 mm, a field of view of 250 mm, a temporal resolution of 5 seconds, 35 dynamic phases, and a total acquisition time of 4 minutes 44 seconds.

### Image Analysis

**Figure 1:**
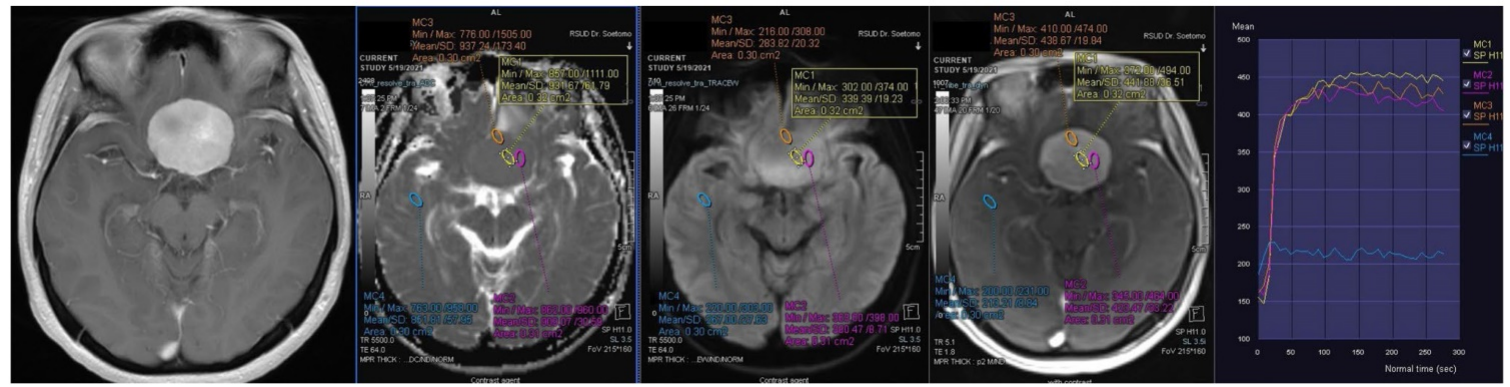
Contrast-enhanced T1-weighted imaging, diffusion weighted imaging, and dynamic contrast enhancement magnetic resonance imaging in a 45-year-old woman with histopathologically confirmed World Health Organization grade I transitional meningioma. The mean apparent diffusion coefficient value was 0.925 x10^-3^mm^2^s^-1^. The time-signal intensity curve showed rapid initial enhancement followed by a plateau phase (Type III).

**Figure 2:**
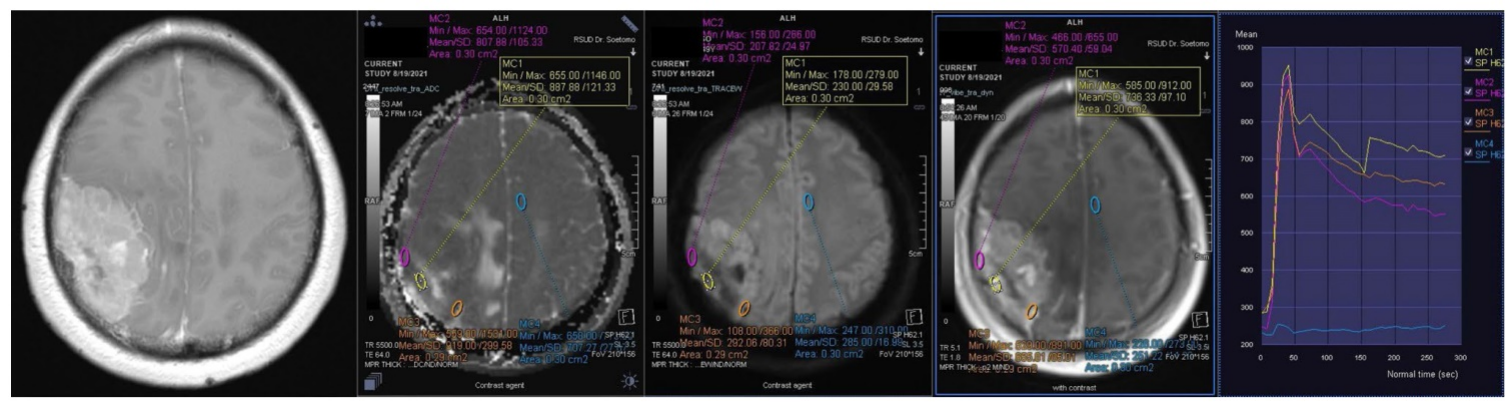
Contrast-enhanced T1-weighted imaging, diffusion weighted imaging, and dynamic contrast enhancement magnetic resonance imaging in a 49-year-old man with histopathologically confirmed World Health Organization grade II atypical meningioma. The mean apparent diffusion coefficient value was 0.871 x10^-3^mm^2^s^-1^. The time-signal intensity curve showed rapid initial enhancement followed by the washout phase (Type IV).

Conventional MRIs (contrast-enhanced, T1-weighted imaging sequences) were utilized to determine the tumor size, location, existence of irregular shape, necrotic/cystic changes, and heterogeneous enhancement. T2 fluid-attenuated inversion recovery sequences were used to evaluate peritumoral edema. The ADC value and DCE parameters were analyzed using a Siemens Syngo software workstation. The ROIs of the ADC maps and DCE were manually placed in the solid part of the tumor, thereby evading necrotic, cystic, hemorrhagic, and large vessels. An ROI of 30-50 mm^2^ was automatically synchronized between the ADC value and DCE and measured three times, following which the mean was obtained (Figures 1 and 2).

**Figure 3:**
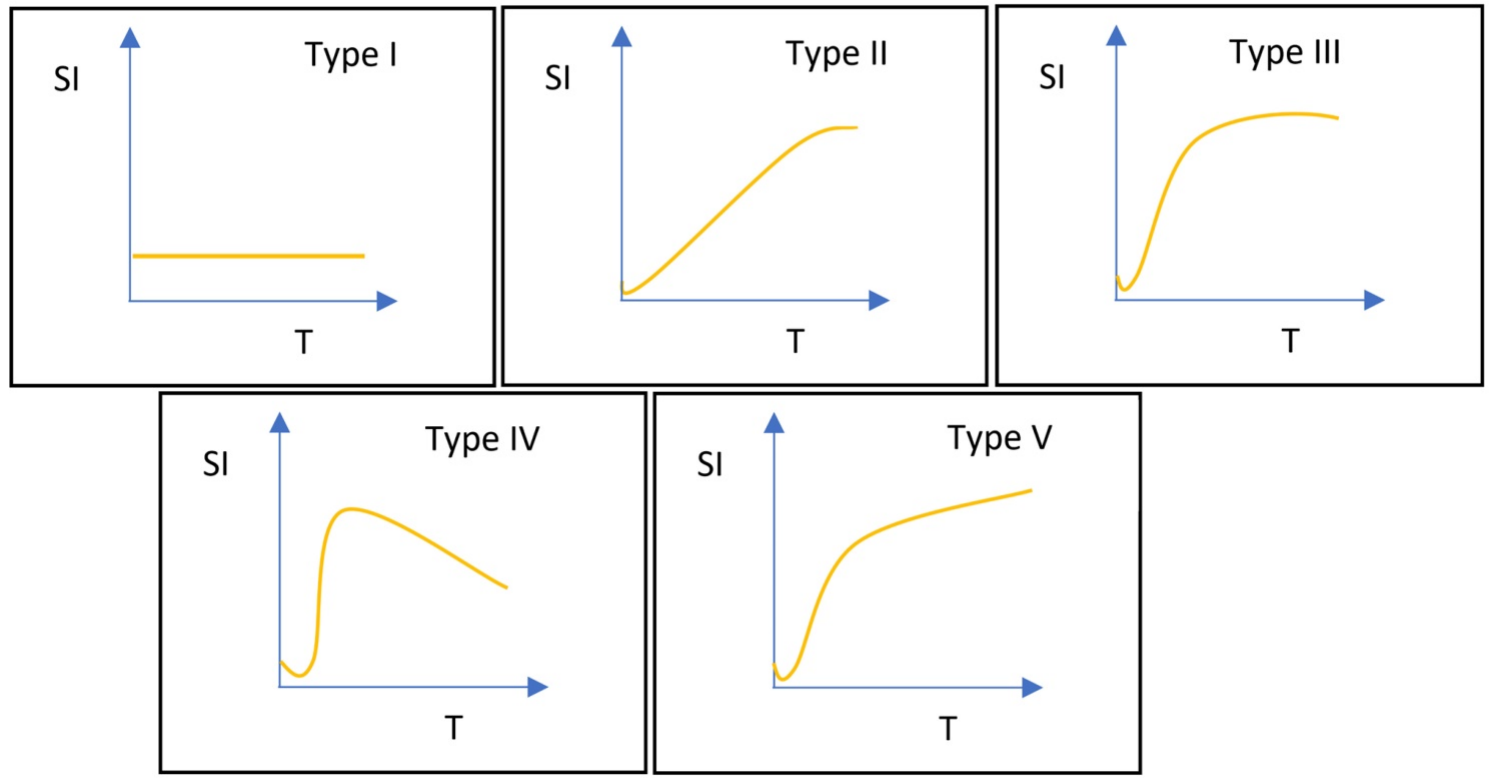
Classifications of time-signal intensity curves. Type I shows no enhancement. Type II shows gradual enhancement. Type III shows a rapid early phase enhancement followed by plateau enhancement. Type IV has a rapid early phase followed by washout enhancement. Type V rapidly rises to a slow enhancement. SI, signal intensity; T, time.

**Figure 4:**
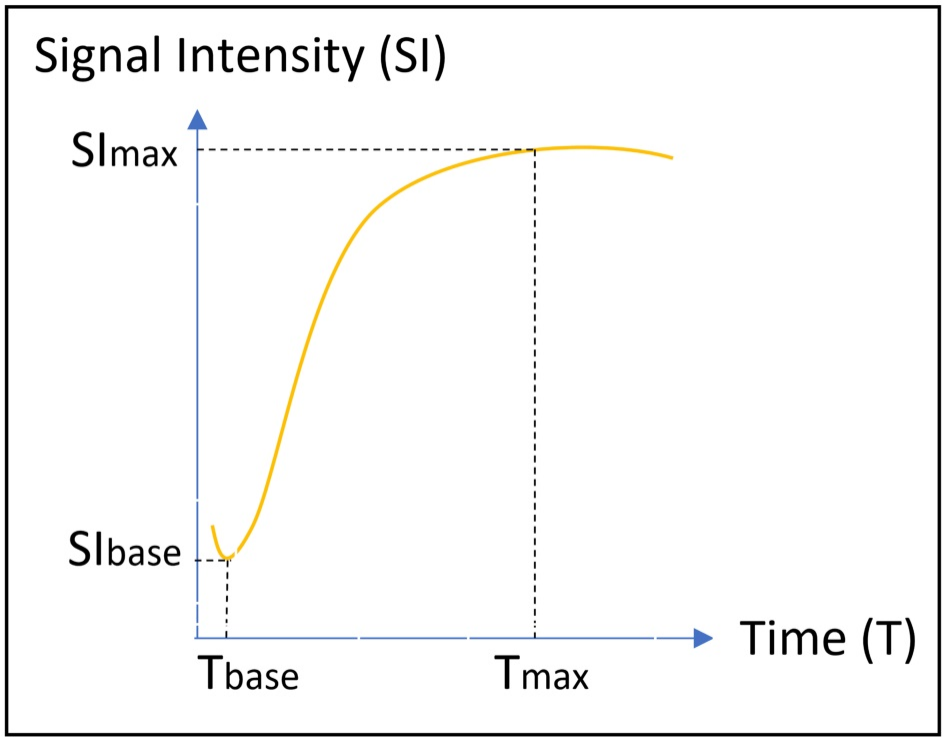
Temporal change in the signal intensity against time in the time-signal intensity curve. SI, signal intensity; T, time.

DCE-MRI data were examined using the Mean-Curve software to obtain the TIC type. According to some radiologists, TIC can be divided into five types (Figure 3). Type I TIC shows no enhancement, type II shows slow enhancement, type III shows a fast initial phase followed by plateau enhancement, type IV is characterized by a fast initial phase followed by washout enhancement, and type V exhibits rapid rises to a slow enhancement. Type IV TIC usually delineates highly vascularized malignant tumors with a small interstitial space 15-17. In addition, we evaluated other DCE parameters, such as SImax, Tmax, MCER, and slope. MCER means the maximum contrast-enhancement rati; slope means the percentage increment in signal intensity per second. The MCER and slope were calculated using the following equations:



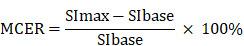





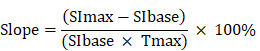



where SIbase denotes the signal intensity before intravenous gadolinium administration, SImax is the maximum value of the signal intensity after contrast agent administration, and Tmax is the time to maximum signal intensity (Figure 4).

All MRI results were analyzed by two board-certified neuroradiologists (initials: SAU and WF) with 25 and 17 years of experience, respectively. The neuroradiologists were blinded to the cases and histopathological grading results. Conventional MRI and TIC type was analyzed by consensus. The agreement between the two neuroradiologists on the calculation of mean ADC values and DCE (SImax, Tmax, MCER, and slope) was appraised by the intraclass correlation (ICC) test. Next, the average value of each parameter was calculated.

### Histopathology Reports

The histopathology reports of surgically resected specimens were evaluated by a neuropathologist(with 18 years of experience). The pathological diagnosis and tumor grading of each specimen was based on the 2016 WHO Classification of Tumors of the Central Nervous System.

### Statistical Analysis

SPSS (version 26.0; IBM Corp., Armonk, New York, USA) was used to conduct all statistical analyses to determine whether the variables differed significantly between HGMs and LGMS. Categorical variables are presented as frequencies (percentages) and were evaluated using the Chi-square or Fisher's exact test. Continuous variables were tested using the Kolmogorov-Smirnov test to evaluate the normality of the distribution. Continuous variables with normal distribution are described as mean ± standard deviation and were analyzed using the independent-sample *t-*test. Continuous variables with skewed distributions were analyzed using the Mann-Whitney *U* test. Statistical significance was set at *p* < 0.05. Receiver operating characteristic (ROC) curve analysis was used to determine the optimal cut-off for significant variables to discriminate between HGMs and LGMS. The diagnostic performance of each significant variable is presented in the form of sensitivity, specificity, positive and negative predictive values, accuracy, and area under the curve (AUC). Subsequently, a multivariate logistic regression analysis was carried out to determine which independent variables were most significant as predictive parameters for differentiating between HGMs and LGMs.

## Results

### Patient Characteristics and Conventional MRI

Thirty-three individuals were included in this retrospective analysis. Twenty-eight (84.8%) patients were pathologically diagnosed with LGMs and five (15.2%) patients with HGMs. The LGM subtypes included 16 transitional meningiomas, seven microcystic meningiomas, three meningothelial meningiomas, one fibrous meningioma, and one metaplastic meningioma. Twenty-eight of the 33 patients (84.8%) included in this study were women. Three of the 28 patients with LGMs and two of the five patients with HGMs were male, indicating a fairly similar distribution of sex in both groups (*p* = 0.15). The mean ages of the patients with LGMs and those with HGMs were also fairly similar (48.21 ± 1.86 vs. 57.6 ± 5.82 years, *p* = 0.07).

The median tumor sizes were also similar between the two groups (32.56 cm^3^ vs. 32.24 cm^3^, *p* = 0.39). The most common locations for HGMs were in the convexity (50%) and skull base (42.9%), while LGMs in this study were located in the convexity. In addition, there were no substantial differences in tumor location between HGMs and LGMs (*p* = 0.23). Five LGMS (17.9%) and two HGMs (40%) had irregular shapes. Six LGMs (21.4%) and two HGMs (40%) demonstrated necrotic/cystic changes. Four LGMs (14.3%) and two HGMs (40%) demonstrated severe peritumoral edema with marked mass effect. Seven LGMs (25%) showed no peritumoral edema, while all HGMs showed variable peritumoral edema. Thirteen LGMS (46.4%) and four HGMs (80%) demonstrated heterogeneous enhancement. No significant differences in shape, necrotic/cystic changes, peritumoral edema, and enhancement patterns were found between LGMs and HGMs (*p* = 0.28, 0.57, 0.56, and 0.33, respectively). Demographic data and conventional MRI parameters are presented in Table 1.

### ADC Value and DCE Parameters

**Figure 5:**
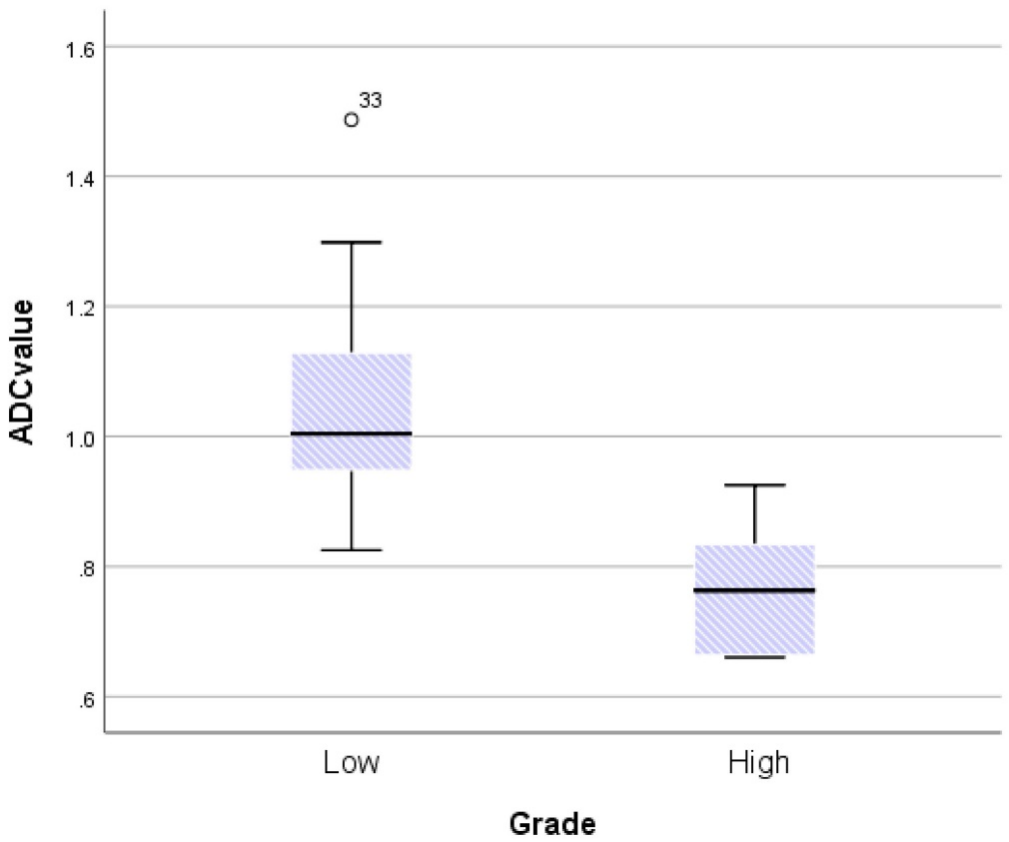
Boxplot comparing apparent diffusion coefficient (ADC) values between World Health Organization (WHO) grade I and grade II/III meningiomas. The upper and lower hinges of the boxes delineate the 75th and 25th percentiles, respectively. The median of apiece distribution is denoted by the line. Whiskers represent the data range. The ADC value was significantly lower in WHO grade II/III meningiomas. *P* = 0.002, calculated by independent-sample *t-test*.

The ICC coefficients for mean ADC value, SImax, Tmax, MCER, and slope were 0.95, 0.97, 0.85, 0.96, and 0.96, respectively. An ICC coefficient of more than 0.75 suggested outstanding reliability. The mean ADC values for LGMs and HGMs are shown in Figure 5. The mean ADC value was noticeably lower in HGMs than in LGMs (0.77 ± 0.5 vs. 1.05 ± 0.28 × 10^-3^ mm^2^s^-1^, *p* = 0.002).

The only TIC types observed in this study were Type III and Type IV. Type IV was present in three of the 28 (10.7%) LGMs and four of the five (80%) HGMs. There was a significant difference in the type of TIC between LGMs and HGMs (*p* = 0.004).

**Figure 6:**
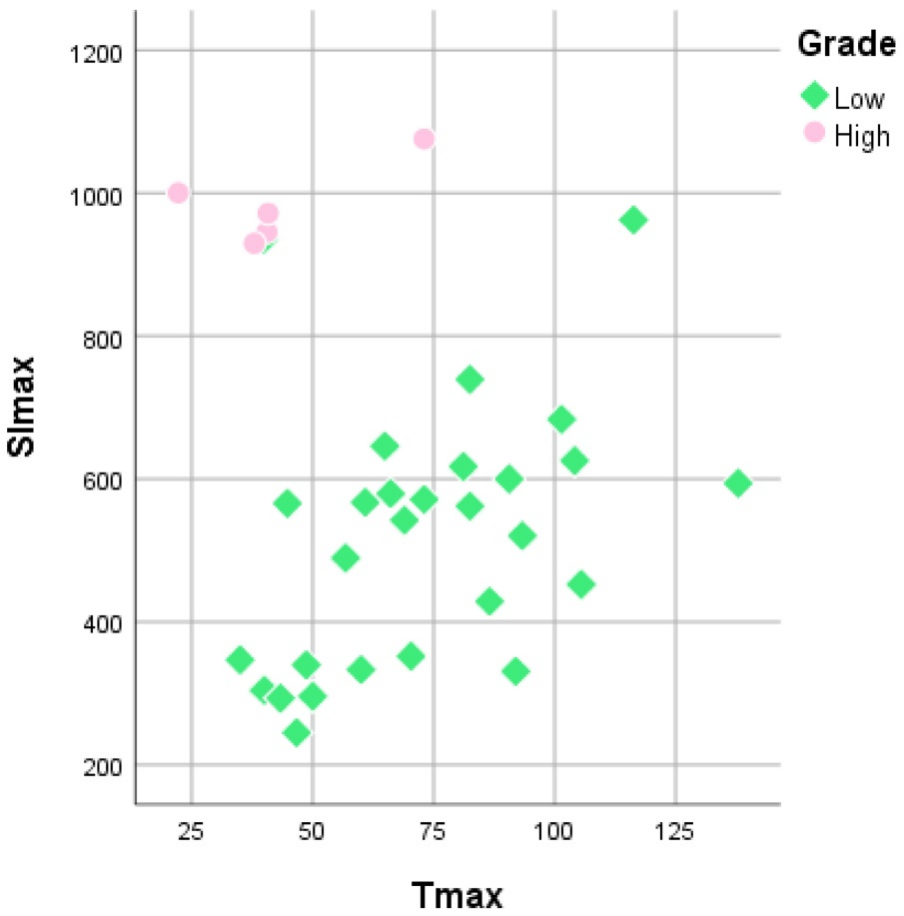
Scatterplot of time to maximum signal intensity (Tmax) versus maximum signal intensity (SImax) between World Health Organization grade I and grade II/III meningiomas. Grade I (green diamonds) and grade II/III (pink rounds) meningiomas are better separated in the SImax than in Tmax.

The mean Tmax of LGMs was 72.96 ± 4.91 s, while that of HGMs was 42.89 ± 8.28 s. There was a substantial difference in the Tmax between LGMs and HGMs (*p* = 0.02). However, a scatter diagram showed that the SI max plots were better separated than the Tmax plots (Figure 6).

**Figure 7:**
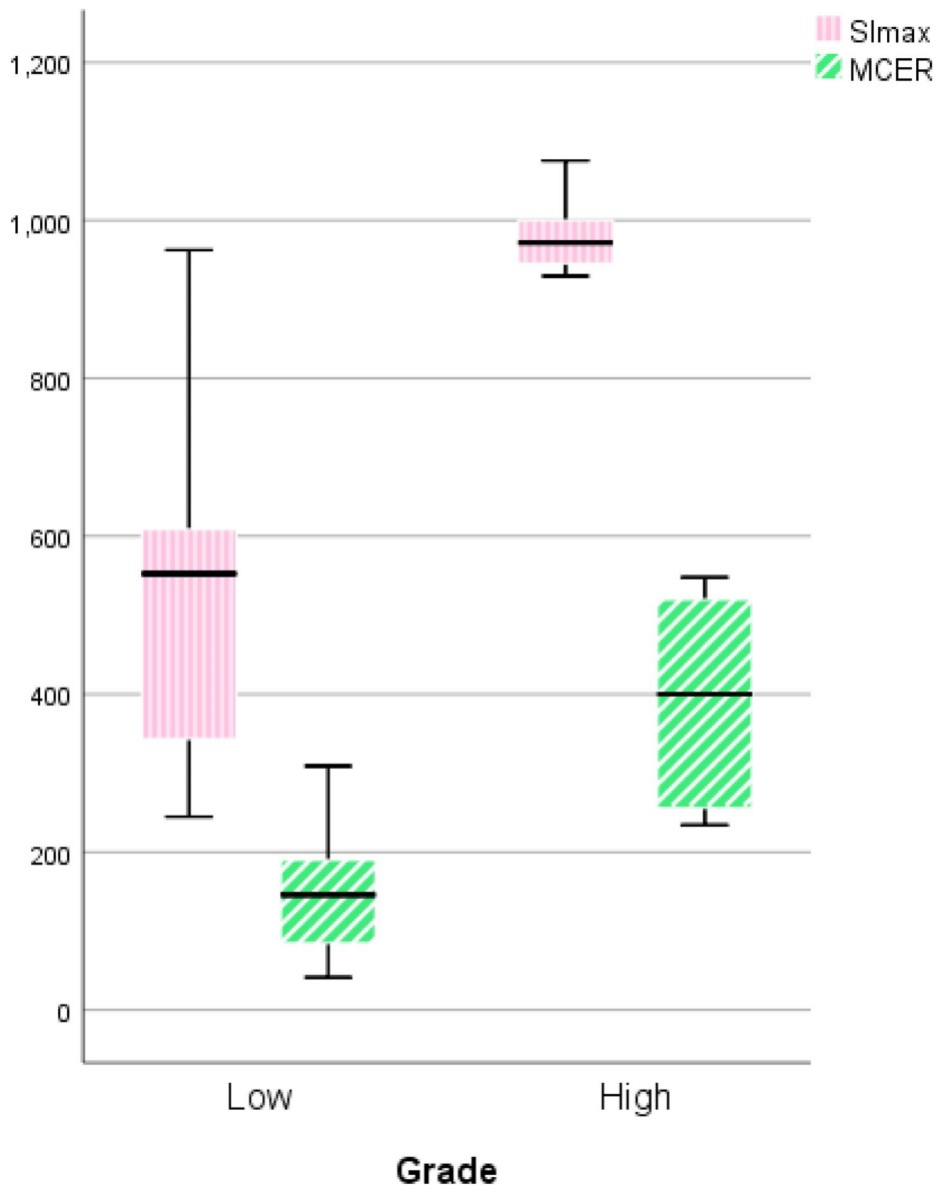
Boxplot comparing the maximum signal intensity (SImax) and maximum contrast enhancement ratio (MCER) between World Health Organization (WHO) grade I and grade II/III meningiomas. The SImax and MCER were higher in WHO grade II/III meningiomas. *P* = 0.000 and *P* = 0.019, respectively, calculated by the independent-sample *t-test*.

**Figure 8:**
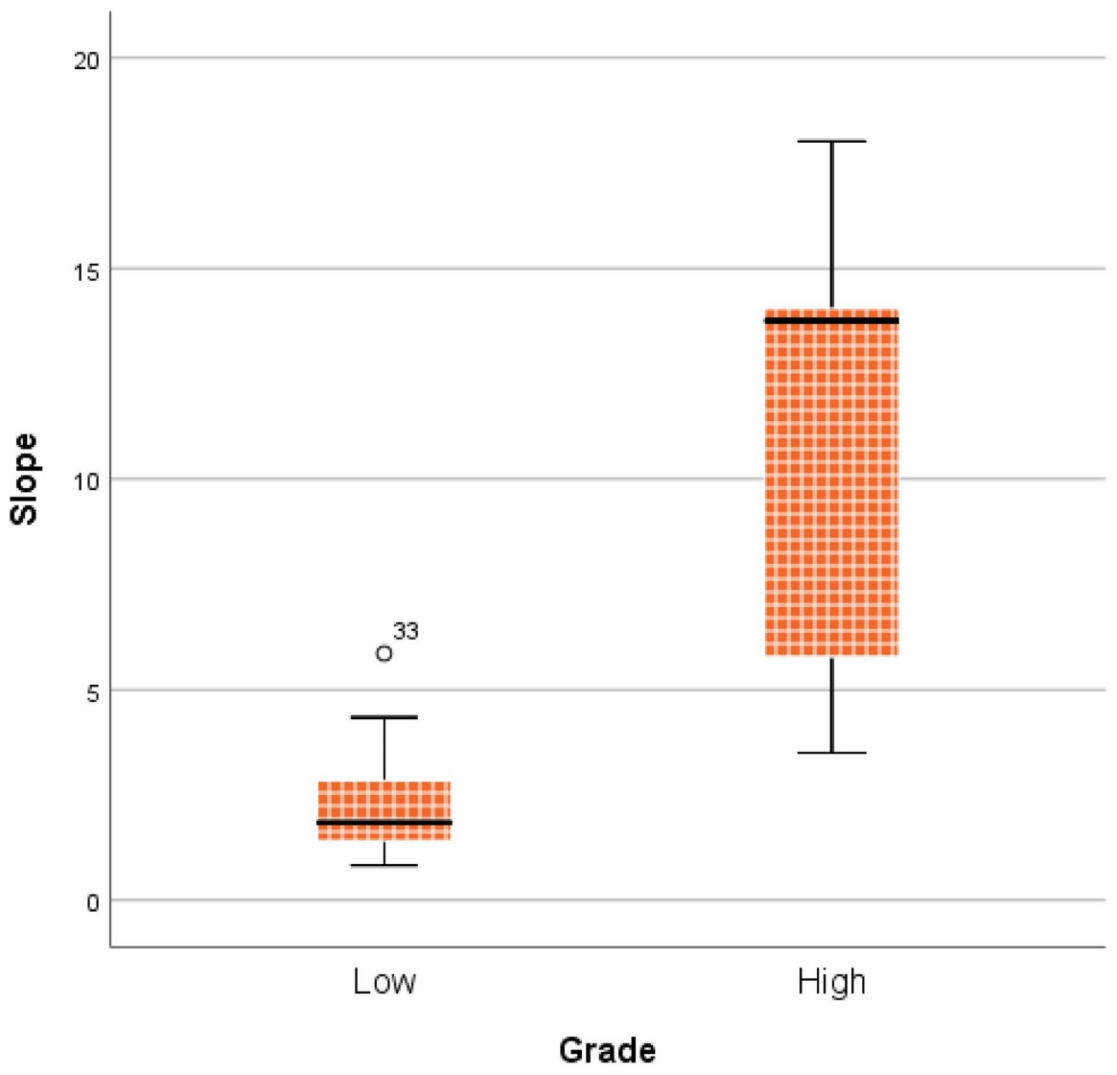
Boxplot comparing slope between World Health Organization (WHO) grade I and grade II/III meningiomas. The median slope was higher in WHO grade II/III meningiomas. *P* = 0.001, calculated by the Mann-Whitney *u* test.

**Figure 9:**
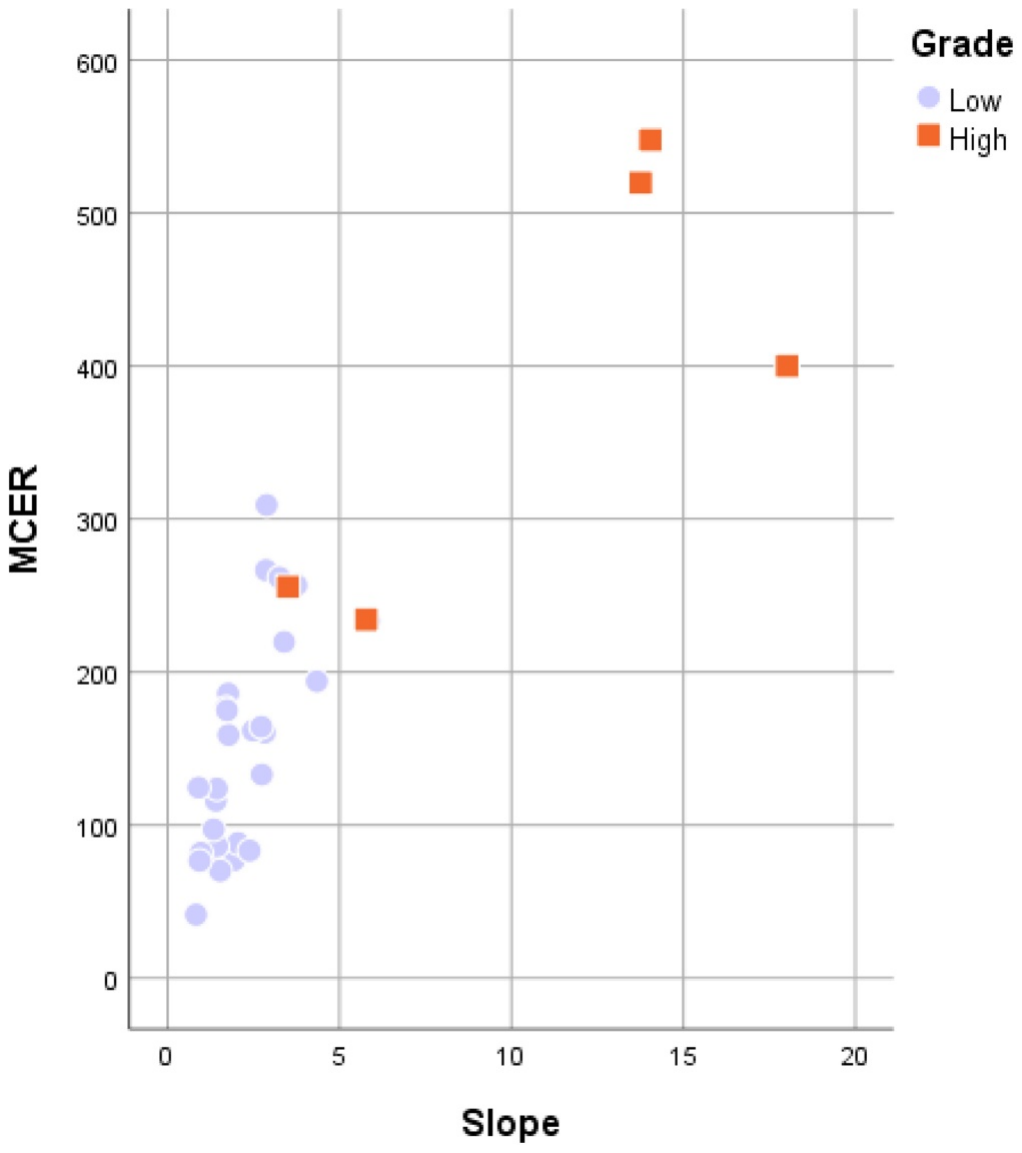
Scatterplot of the maximum contrast enhancement ratio (MCER) versus slope between World Health Organization grade I and grade II/III meningiomas. Grade I (purple rounds) and grade II/III (orange rectangles) meningiomas are both well separated in the MCER and slope.

Figure 7 shows the comparison between LGMs and HGMs in terms of SImax and MCER. The mean SImax was significantly higher in HGMs than in LGMs (984.43 ± 25.76 vs. 518.66 ± 34.55, *p* < 0.001). In addition, Figure 6 shows that the SImax plot was well separated. The mean MCER in HGMs (391.49 ± 64.82%) was significantly higher than in LGMS (150.01 ± 13.43%, *p* = 0.02). Figure 8 shows that the median slope in HGMs (13.76% per second) was also substantially higher than that in LGMs (1.85% per second, *p* = 0.001). Moreover, the MCER and slope were well separated in the scatter plot (Figure 9). The mean ADC values and DCE parameters are presented in Table 2.

**Figure 10:**
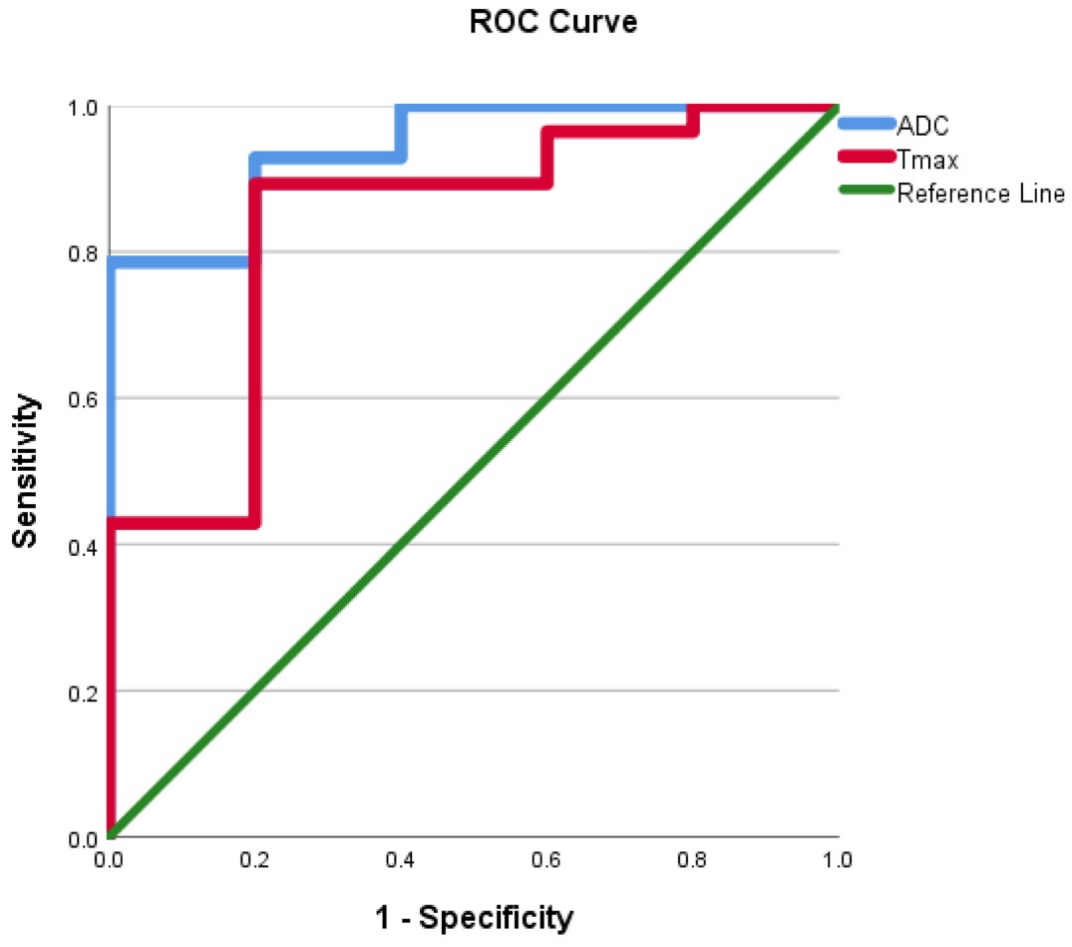
Receiver operating characteristic curve for use of the apparent diffusion coefficient value and time to maximum signal intensity in differentiating World Health Organization (WHO) grade II/III meningiomas from WHO grade I meningiomas. ADC, apparent diffusion coefficient; Tmax, time to maximum signal intensity.

**Figure 11:**
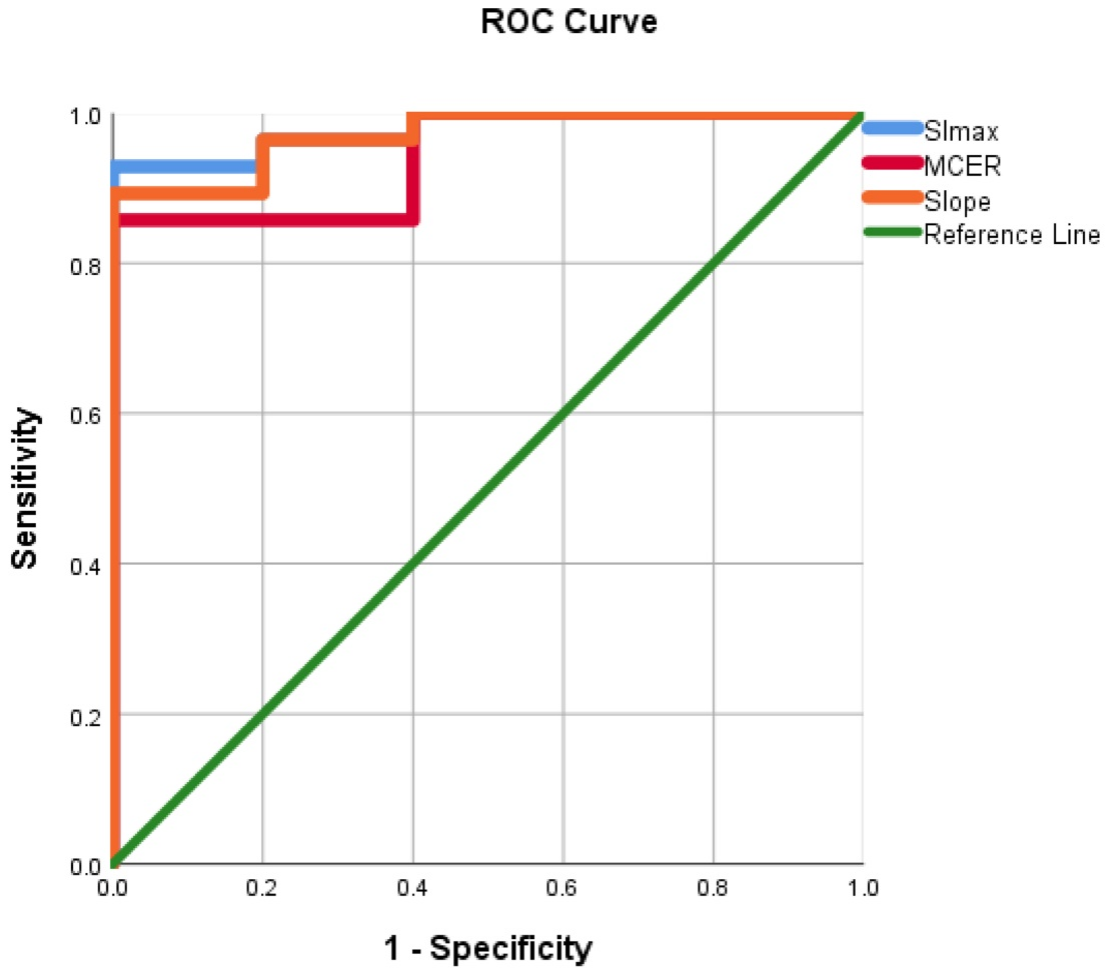
Receiver operating characteristic curve for use of maximum signal intensity, maximum contrast enhancement ratio, and slope in differentiating World Health Organization (WHO) grade II/III meningiomas from WHO grade I meningiomas. SImax, maximum signal intensity; MCER, maximum contrast enhancement ratio.

ROC curves were evaluated using the mean ADC value, SImax, Tmax, MCER, and slope as predictors for HGM diagnosis as opposed to LGMs. ROC analyses, as shown in Figures 10 and 11, showed that the AUC of the mean ADC value, SImax, Tmax, MCER, and slope had excellent diagnostic performance (AUC = 0.94, 0.98, 0.84, 0.94, and 0.97, respectively). Mean ADC values < 0.88 × 10^-3^ mm^2^s^-1^ differentiated HGMs from LGMs with a sensitivity and specificity of 80% and 92.8%, respectively. SImax and slope provided the best diagnostic values of all the DCE parameters. SImax > 0.98 and slope > 5% per second both had a sensitivity of 80% and specificity of 96.4% in distinguishing HGMs from LGMs. The ROC curve results and diagnostic values are presented in Table 3. Subsequently, a multivariate logistic regression analysis was carried out to determine the most significant predictor for meningioma grade. The mean ADC value was found to be the most powerful predictor of meningioma grade (*p* < 0.001).

## Discussion

Although HGMs are less common than LGMs, they have higher rates of recurrence. Atypical meningiomas account for 5-15% of cases, with recurrence rates of 30-40%, while anaplastic or malignant meningiomas account for 1-3% of cases, with a recurrence rate of 50-80% 1. Therefore, the capacity to diagnose HGMs through noninvasive examination is desirable to optimize treatment management for individual patients. This study aimed to determine the ability of conventional MRI, DWI, and especially DCE, to distinguish HGMs from LGMs.

We identified no significant differences in the sex and age of patients with LGMs and HGMs; however, all meningiomas were predominant in women. Women of reproductive age with a family history of breast cancer have an increased likelihood of developing meningiomas 1. Similar to a previous study, LGMs had a slight predominance in women 18. The risk of developing meningiomas is associated with hormonal exposure. Older patients also had a slightly higher risk of developing a higher-grade meningioma, consistent with a previous study 19. It is debatable if age is a risk factor for HGMs.

There is a crossover between LGMs and HGMs in conventional MRI. Tumor size, location, shape, necrotic/cystic changes, peritumoral edema, and enhancement pattern between the two groups were not statistically significantly different, which is in line with previous studies 20-24. HGMs tend to have an unclear border between the tumor and normal brain parenchyma. The irregular shape of meningiomas is caused by intratumoral pressure discrepancies. Unclear borders and irregular shapes have been reported as signs of tumor aggressiveness 23. Meningiomas frequently have homogenous enhancement. Heterogeneous enhancement following gadolinium contrast agent administration is associated with unequal proliferating tumor cell distribution or even ischemic necrosis, which are biological hallmarks of high-grade tumors 24. Since the unintentional leakage of plasma, fluid, and other molecules via a compromised blood-brain barrier causes peritumoral edema, the appearance of peritumoral edema may be related to HGMs 23. However, conventional MRI features are not reliable in distinguishing meningioma grades, although a previous study found that tumor volume, necrosis, peritumoral edema, and location were predictive of HGMs 25.

DWI is frequently used to assess brain tumors, particularly for tumor categorization, histological grading, and therapy monitoring. ADC values have been used in several studies to determine meningioma consistency and grade 6,26. According to certain research, there is no statistically significant difference between the ADC values of LGMs and HGMs 27,28. However, Surov et al. reported in an analysis of 49 patients with meningiomas that the mean ADC value of HGMs (0.8 × 10^-3^ mm^2^s^-1^) was significantly lower than that of LGMs (0.96 × 10^-3^ mm^2^s^-1^, *p* = 0.006) 4. Other authors have also reported that HGMs have lower ADC values than LGMS 6,7,29,30.

Our study suggested a mean ADC value cut-off ≤ 0.87 × 10^-3^ mm^2^s^-1^ to differentiate HGMs from LGMs, with an AUC of 0.94. This result is similar to other studies. Surov et al. suggested a threshold ADC value of less than 0.85 × 10^-3^ mm^2^s^-1^, with a sensitivity of 72.9%, specificity of 73.1%, and AUC of 0.8 4. Tang et al. suggested an ADC cut-off value of greater than 0.85 × 10^-3^ mm^2^s^-1^ to predict LGMs 30, while Xiaoai et al. proposed an ADC cut-off value of 0.86 × 10^-3^ mm^2^s^-1^ for differentiating microcystic meningiomas from atypical meningiomas 22.

Numerous concepts have been proposed to explain the low ADC value in high-grade tumors. The speed of water molecule diffusion in tumor tissue depends on the ratio of intracellular to extracellular spaces. HGMs have a low ADC value owing to the restricted diffusion area of water molecules caused by high tumor cellularity and decreased extracellular space in the tumor tissues 7,22. Moreover, other factors contribute to reduced water molecule diffusion in HGMs, such as greater necrotic area, higher mitotic cellular proliferation, and conspicuous nuclei as opposed to the cytoplasm. However, not all HGMs contain a significant amount of tumor cellularity. In our study, one case of atypical meningioma showed augmented water molecule diffusion compared with other HGMs (mean ADC value = 0.92). A previous study hypothesized that some HGMs tend to develop from meningiomas with a more benign appearance, therefore demonstrating the characteristics of benign meningiomas 7.

DSC and DCE are perfusion MRI techniques that use contrast agents to study tumor microvasculature. However, meningiomas are highly vascular tumors without a blood-brain barrier; therefore, measurement of relative cerebral blood flow in DSC-MRI is not appropriate 31. The signal intensity-time curve of DSC-MRI shows immediate negative contrast enhancement due to the leakage of contrast media in the absence of the blood-brain barrier 7. However, DCE-MRI can semi-quantitatively reflect tissue physiology, which allows for the evaluation of dynamic changes in tumor tissue perfusion, local endothelial tissue permeability, and interstitial fluid. High perfusion and highly permeable endothelial tissues show early enhancement and more intense contrast uptake 17. To the best of our knowledge, no study has investigated DCE parameters, such as TIC, SImax, Tmax, MCER, and slope, for grading meningiomas. The results of this study not only demonstrate strong predictive factors but also have significant implications for clinical treatment.

The rapid diffusion of the contrast agent into the tissue during the initial transit through the capillaries is caused by the significant concentration gradient between the intravascular and interstitial regions. In normal tissues, roughly half of the circulating contrast agent diffuses from the blood into the extravascular compartment during the initial phase. The diffusion rate of the contrast agent quickly decreases after the initial phase because the concentration of the recirculating contrast agent has dropped owing to dilution in the circulation and partial deposition in the interstitial space all across the tissues. Early washout may occur in the first minutes following bolus injection in extremely vascularized tumor tissues with a short interstitial space 16.

Our study identified statistically significantly different types of TIC in HGMs and LGMs. Type IV TIC was more likely to be observed in HGMs. Type IV TIC is defined by a fast initial phase followed by washout enhancement, which delineates highly vascularized tumors with a tiny interstitial space. In the dynamic contrast study, the initial contrast flow was employed to assess tumor vascularization and tissue perfusion. Tumors with high vascularization and increased capillary permeability tend to absorb contrast earlier and more intensely than less vascularized tissues. One case of HGM in this study showed type III TIC. This was the aforementioned case having a high ADC value and benign morphology. Costa et al. also reported that type IV TIC is more commonly seen in malignant tumors; however, it can also be found in some benign tumors 17.

Furthermore, our study found that the Tmax was lower in HGMs than in LGMs, with an optimal cut-off of 42 s. Malignant tumors tend to exhibit faster contrast enhancement than benign tumors. SImax and MCER of greater than 940.2% and 943%, respectively, were estimated as the optimal cut-offs for differentiating between HGMs and LGMs. MCER is the total volume accumulation of contrast uptake in the tumor vascular and interstitial spaces; therefore, it represents the maximum increment between pre- and post-contrast injection. In our study, SImax showed better specificity than MCER (96.4 vs. 85.7%). This may be due to the different SIbases noted in each tumor. Tumors with a lower SIbase may have a higher MCER, although they do not show a greater SImax. The presence of contrast agents in the intravascular region, as well as the increasing contribution of contrast agents in the extracellular space, explains the rapid increase in signal intensity shortly after injection. An extracellular contrast agent is swiftly removed from the intravascular space, and extracellular space equilibrium is achieved 16. The extracellular space is gradually washed out, which corresponds to the progressive decline in signal intensity over time. HGMs tend to absorb contrast more intensely than LGMs due to augmented capillary permeability. A slope of greater than 5% per second was determined as the optimal threshold for differentiating between HGMs and LGMs (sensitivity = 80%; specificity = 96.4%; and accuracy = 93.9%). ROC analysis showed that the AUC of the slope was 0.971. The slope is the percent increase in signal intensity per second; thus, it represents the early contrast enhancement shown in a TIC and therefore may semi-quantitatively calculate the physiological perfusion of the tumor tissues. According to the present theory, vascular permeability and the diameters of endothelial gap junctions are significantly related to the tumor grade.

A previous study used quantitative DCE-MRI to differentiate between atypical and typical meningiomas. The study showed that *K*trans could be used to differentiate between the two groups (*p* < 0.01), while other parameters such as *K*ep, *V*p, and *V*e did not differ significantly between the two groups 32. HGMs are associated with a higher permeability than LGMs, as shown by the higher *K*trans in HGMs. The lack of a correlation between tumor grade and *K*ep shows that the rate of transfer from the intravascular space to the extravascular space is more relevant than the absolute indices of contrast material leakage 32. Both quantitative and semiquantitative parameters of DCE-MRI show that HGMs have higher vascularization and capillary permeability; therefore, they tend to have earlier and greater contrast enhancement than LGMs.

Our study has some limitations. First, it was retrospective, and only five of the 33 patients included in this study had HGMs. Second, quantitative DCE-MRI analysis was not performed. Third, the diameter of the ROI was quite small. Fourth, the population was heterogeneous and included five subtypes of LGMs.

In conclusion, ADC values and DCE-MRI parameters (TIC, SImax, Tmax, MCER, and slope) are potential predictors for differentiating HGMs from LGMs.

## Figures and Tables

**Table 1:** Demographic data and conventional Magnetic Resonance Imaging of low-grade and high-grade meningiomas

Variable	Low-grade	High-grade	*p* value
Sex			0.15
Female	25 (89.3%)	3 (60%)	
Male	3 (10.7%)	2 (40%)	
Age, years			0.07
Mean	48.21 ± 1.86	57.6 ± 5.82	
Tumor size, cm^3^			0.39
Median	32.56	32.24	
Location			0.23
Convexity	14 (50%)	5 (100%)	
Skull base	12 (42.9%)	0	
Falx	1 (3.6%)	0	
Posterior fossa	1 (3.6%)	0	
Irregular shape			0.28
Yes	5 (17.9%)	2 (40%)	
No	23 (82.1%)	3 (60%)	
Necrotic/Cystic changes			0.57
Yes	6 (21.4%)	2 (40%)	
No	22 (78.6%)	3 (60%)	
Peritumoral edema			0.56
Yes	21 (75%)	5 (100%)	
No	7 (25%)	0	
Heterogenous enhancement			0.33
Yes	13 (46.4%)	4 (80%)	
No	15 (53.6%)	1 (20%)	

**Table 2:** ADC values and DCE parameters of low-grade and high-grade meningiomas

Variable	Low-grade	High-grade	*p* value
ADC value, x10^-3^ mm^2^s^-1^			0.002
Range	0.82-1.49	0.66-0.92	
Mean	1.05 ± 0.28	0.77 ± 0.5	
TIC			0.004
Type III	25 (89.3%)	1 (20%)	
Type IV	3 (10.7%)	4 (80%)	
SImax			< 0.001
Range	244.85-962.24	929.50-1075.68	
Mean	518.66 ± 34.55	984.43 ± 25.76	
Tmax, seconds			0.02
Range	35-137.92	22.2-73.02	
Mean	72.96 ± 4.91	42.89 ± 8.28	
MCER, %			0.02
Range	41.34-309.11	234.31-547.75	
Mean	150.01 ± 13.43	391.49 ± 64.82	
Slope, % per second			0.001
Range	0.83-5.86	3.5-18.02	
Median	1.85	13.76	

ADC, apparent diffusion coefficient; DCE, dynamic contrast enhancement; TIC, time-signal intensity curve; SImax, maximum signal intensity; Tmax, time to maximum signal intensity; MCER, maximum contrast enhancement ratio

**Table 3:** ROC results of ADC values and DCE parameters for distinguishing high-grade meningiomas from low-grade meningiomas.

Variable	AUC	Cut-off	Sensitivity	Specificity	PPV	NPV	Accuracy
ADC value	0.94	0.87	80	92.8	66.6	96.3	90.9
SImax	0.98	940.2	80	96.4	80	96.4	93.9
Tmax	0.84	42	80	89.3	57.1	96.1	87.8
MCER	0.94	245	80	85.7	50	96	84.8
Slope	0.97	5	80	96.4	80	96.4	93.9
TIC	0.85	Type IV	80	89.3	57.1	96.1	87.8

ROC, receiver operating characteristic; ADC, apparent diffusion coefficient; DCE, dynamic contrast enhancement; SImax, maximum signal intensity; MCER, maximum contrast enhancement ratio; AUC, area under the curve; PPV, positive predictive value; NPV, negative predictive value
